# Antioxidant and Anti-Inflammatory Effects of NCW Peptide from Clam Worm (*Marphysa sanguinea*)

**DOI:** 10.4014/jmb.2003.03050

**Published:** 2020-07-17

**Authors:** Young Ran Park, Chan-Il Park, Yunjo Soh

**Affiliations:** 1 Department of Dental Pharmacology, School of Dentistry, Jeonbuk National University, Jeonju 54896, Republic of Korea; 2 Department of Marine Biology and Aquaculture, College of Marine Science, Gyeongsang National University, Tongyeong 52828, Republic of Korea; 3 Department of Pharmacology, School of Pharmacy and Institute of New Drug Development, Jeonbuk National University, Jeonju 54896, Republic of Korea

**Keywords:** Novel peptide, clam worm, antioxidant, anti-inflammation

## Abstract

Clam worms (*Marphysa sanguinea*) are a rich source of bioactive components such as the antibacterial peptide, perinerin. In the present study, we explored the physiological activities of a novel NCWPFQGVPLGFQAPP peptide (NCW peptide), which was purified from clam worm extract through high-performance liquid chromatography. Tandem mass spectrometry (MS/MS) revealed that NCW was a new peptide with a molecular weight of 1757.86 kDa. Moreover, NCW peptide exhibited significant antioxidant effects, causing a 50% inhibition of DPPH radical at a concentration of 20 μM without showing any cytotoxicity. These were associated with a reduction in the activity of catalase (CAT), superoxide dismutase (SOD), glutathione peroxidase (GSH-Px), and malondialdehyde (MDA) in LPS-stimulated RAW264. 7 cells. Furthermore, NCW peptide exhibited anti-inflammatory effects in LPS-stimulated RAW264.7 macrophages via inhibition of the abnormal production of pro-inflammatory cytokines including nitric oxide (NO), inducible nitric oxide synthase (iNOS), and cyclooxygenase-2 (COX-2). These anti-inflammatory effects of NCW peptide were associated with the inhibition of interleukin-1β (IL-1β) and tumor necrosis factor-α (TNF-α). Our results therefore suggest that this novel NCW peptide with antioxidant and anti-inflammatory effects could be a good therapeutic agent against inflammation-related diseases.

## Introduction

Inflammation has been well established as a localized response of tissue to irritation, injury, or infection characterized by pain, redness, and swelling. Commonly, inflammation is triggered by the generation of specific cytokines or chemokines and is distinguished by the gathering of leukocytes to the damage site. Lipopolysaccharide (LPS), derived from the outer membrane (OM) of gram-negative bacteria, leads to the activation of reactive oxygen species (ROS) and oxidative stress [1, 2]. Previous studies have shown that LPS can activate nuclear factor-kappa B (NF-κB), a major nuclear transcription factor that controls various inflammatory regulators such as tumor necrosis factor-α (TNF-α), interleukin-1β (IL-1β), inducible nitric oxide synthase (iNOS) and cyclooxygenase-2 (COX-2) [3, 4].

During inflammation, oxidative stress also leads to high levels of ROS and activation of malondialdehyde (MDA) and decreases production of anti-oxidant enzymes, such as catalase (CAT), superoxide dismutase (SOD) and glutathione peroxidase (GSH-Px) [5]. Clinically, high levels of oxidative damage have been positively associated with cardiovascular diseases, diabetes, and chronic inflammation [6]. Thus, inhibiting levels of inflammatory factors and oxidative stress may help in treating various inflammation-related diseases.

Earthworms have a high density of nutritional content which is of soil-based origin [7]. Recent findings on earthworms have shown them to possess antipyretic, anti-spasmodic, detoxic, diuretic, anti-hypertensive, anti- allergic, anti-asthmatic, anti-oxidation, anti-microbial, anti-cancer and anti-inflammation properties [8].

Thus far, antimicrobial peptides have been identified from some *Polychaetes* species, including *Arenicola marina*, *Perinereis aibuhitensis*, and *Nereis diversicolor*. Lectin (chitin-binding protein) and the antimicrobial peptide arenicin have been extracted from the coelomocytes or fluid of the marine polychaeta lugworm *A. marina*. Moreover, the antimicrobial peptide perinerin has been purified from the Asian marine clamworm, *P. aibuhitensis*, and the antimicrobial peptide hedistin was identified from the coelomocytes of *N. diversicolor* [9]. However, there is relatively little information on the antioxidant capacity and anti-inflammatory activities of the clam worm and its cellular mechanism for modulating inflammation and oxidants in host defense.

In our previous study, we reported that clam worm extracts have antioxidant and anti-inflammatory effects with treatment of peptidoglycan [10]. Clam worm also exhibited strong antioxidant and anti-inflammatory activities when compared with earthworm extract in LPS-induced RAW264.7 cells [11].

In this study, we investigated whether NCW peptide, a novel bioactive peptide from worm (*Marphysa sanguinea*) extract, is involved in antioxidant and anti-inflammatory effects in mouse macrophage. To identify NCW peptide from clam worm extract, we used HPLC and MS/MS analysis and isolated the NCW peptide. We found that NCW peptide was a novel peptide extracted from clam worm and there was no cytotoxicity in LPS-induced Raw264.7 cells. Furthermore, our results showed that NCW peptide markedly suppressed the generation of MDA and increased activity of CAT, SOD, and GSH-Px in LPS-stimulated RAW264.7 cells. Moreover, NCW peptide significantly inhibited the activity of pro-inflammatory cytokines such as IL-1β and TNF-α by blocking the NO, iNOS and COX-2 expression in LPS-stimulated RAW 264.7 cells. Thus, these results suggest that NCW peptide may be a new therapeutic candidate to treat a variety of inflammation-related diseases.

## Materials and Methods

### Tissue Extraction and Reverse-Phase HPLC

An NCW peptide was isolated from the marine clam worm, *Marphysa sanguinea*. Adult clam worms (~200 g) were absorbed in clean, mildly cold, artificial sea water with 2.7-3% marine salt for 48 h to remove gastrointestinal metabolites and filth. Clam worms were maintained in frozen state at -80°C by deep freezer (Ilshin Rab Co., Ltd.). Once in a frozen state, the clam worms were added to four volumes of pre-heated 1% acetic acid (HAc) (1:4, v/v) and boiled for 5 min to inhibit proteolytic enzyme activity. The boiled tissue was then completely homogenized (Speed #6, T10 Basic Ultra-Turrax, USA) on wet ice for 5 min. The homogenate was centrifuged (15,000 g, 30 min, 4°C), and the supernatant was stored at -70°C until use. The peptides in the supernatant were then subjected to reverse-phase concentration using a Sep-Pak C18 cartridge (Waters Associates, USA). The active fractions were pooled and further purified by reverse-phase HPLC on a Delta Pak C18 column (Waters Associates) with a linear gradient of 0-60% acetonitrile containing 0.1% (v/v) trifluoroacetic acid over 60 min at a flow rate of 1 ml/min. The column effluent was detected by its UV absorption at 225 nm, and the absorbance peaks were collected and used for antimicrobial activity analysis after lyophilization.

### Mass Spectrometry (MS) and Peptide Sequencing

Peptide mixtures were analyzed by mass spectrometer (UPLC-Q-TOF) (micrOTOF-Q III, Bruker). The peptides were separated in a column (C18, 5 μm, 32 × 150 mm) and eluted with a linear gradient of 10-45% solvent B (100% acetonitrile, 0.1% FA in water) in Solvent A (0.1% FA in water) at a flow rate of 0.1 ml/min. All MS/MS analyses were carried out using the positive ion mode between m/z 50 and 2,000. MS/MS ions were analyzed by Data Analysis (4.0) to confirm the peptide sequence. The sequence of the identified peptides was confirmed by BioTool (3.2).

### Cell Culture and Treatment of LPS

RAW264.7 cells were obtained from the American Type Culture Collection (ATCC, USA) and were maintained with Dulbecco’s Modified Eagle’s Medium (DMEM, Gibco, Waltham, MA, USA) supplemented with 10% heat- inactivated fetal bovine serum (FBS, Gibco), 100 U/ml penicillin (Gibco), and 100 μg/ml streptomycin (Gibco). The cells were cultured in a humidified atmosphere of 5% CO_2_ at 37°C. For stimulation with LPS, RAW264.7 cells were pre-incubated in the presence or absence of indicated concentrations of NCW peptide for 2 h in serum-free media. Then, the cells were incubated with LPS (2 μg/ml) PBS for 20 h in conditioned medium.

### DPPH Radical Scavenging Activity

The DPPH radical scavenging activity was modified by l,1-diphenyl-2-picryl-hydrazyl (DPPH) according to the previously exhibited methods [12]. Briefly, the DPPH reagent was dissolved in methanol for a solution concentration of 80 μl/ml. To determine the scavenging activity, 100 μl of DPPH reagent was added with 100 μl of sample in a 96-well microplate, which was incubated at 37°C for 15 min. The absorbance was measured at 517 nm using a microplate reader, and 100% methanol was used as a blank (A0). The decrease in absorption of DPPH solution is calculated as follows:

% of absorption decreased = (A0-A1) ×100/A0

Vitamin C was used as a positive control and the antioxidant was evaluated based on this IC_50_ value. All samples were carried out in triplicate.

### MTT Assay

Cell viability was determined by MTT [3-(4,5-dimethylthiazol-2-yl)-2,5-diphenyltetrazolium bromide] assay. In brief, 5 × 10^3^ cells/well of RAW264.7 cells were incubated with various concentrations of NCW peptide for 24. Media containing 100 μg/ml of MTT solution were added to cells for 2 h at 37°C. The conditioned media were then removed and purple formazan crystals were solubilized with DMSO. The absorbance was determined at 540 nm using a spectrophotometer and all samples were assayed in triplicate.

### Measurements of Antioxidant Activity

To determine the concentration of released levels of CAT, SOD, GSH-Px, and MDA, RAW 264.7 cells were induced with various concentrations of NCW peptide for 2 h and treated with LPS (2 μg/ml) for 20 h. The catalase activity was analyzed according to the Aebi method [13] and calculated by the change of the absorbance at 340 nm. The amount of SOD (Oxis Research, USA) and GSH-Px (Oxis International, Inc., USA) in cultured medium were quantified according to the manufacturer’s instructions, respectively. MDA was determined by the method of Buege and Aus [14]. All samples were assayed in triplicate.

### Nitric Oxide (NO) Assay

The concentration of nitric oxide in the culture supernatant was analyzed using the Griess reagent [15]. In brief, 150 μl of the culture supernatant was mixed with 50 μl of Griess reagent (1% sulfanilamide and 0.1% naphthylethylene diamine in 2.5% phosphoric acid solution) and incubated at room temperature for 10 min. The absorbance at 540 nm was measured using a spectrophotometer. Nitrite production in each sample was determined from NaNO_2_ serial dilution standard curve. All samples were assayed in triplicate

### Preparation of Cell Extract

To prepare whole cell lysates, cells were harvested and homogenized in lysis buffer (50 mM Tris–HCl, 150 mM NaCl, 1% NP40, 0.25% sodium deoxycholate, 1 mM EDTA, 1 mM Na_3_VO_4_, 1 mM phenylmethyl-sulfonyl fluoride (PMSF), 1 μg/ml leupeptin, 1 μg/ml pepstatin, 5 μg/ml aprotinin, and 20 mM NaF). The suspension was centrifuged for 15 min and the supernatant was collected as the whole-cell extract.

### Western Blot Analysis

Equal amounts of proteins were separated by 8-10% sodium dodecyl sulfate-polyacrylamide gel electrophoresis (SDS-PAGE) and transferred to a polyvinylidene difluoride membrane (PVDF). Membranes were blocked with a solution of 5% non-fat dry skim milk in TTBS at 17°C for 1 h, and then primary antibodies were probed for overnight. Horseradish peroxidase-conjugated anti-rabbit or anti-mouse were used as secondary antibodies at 17°C for 2 h. Blot images were detected using ECL western blot detection reagent (Amersham Biosciences, USA) and X-ray film.

### Measurement of Pro-Inflammatory Cytokines

To determine the levels of pro-inflammatory cytokines, RAW264.7 cells were pre-incubated with NCW peptide for 30 min and then stimulated with LPS (2 μg/ml) for 8 h or 24 h. Concentrations of tumor necrosis factor-α (TNF-α) and interleukin-1β (IL-1β) in the culture supernatants were analyzed using a commercially available mouse TNF-α and IL-1β ELISA kit (R&D Systems, USA) according to the manufacturer’s instructions. All samples were assayed in triplicate.

### Statistical Analysis

Statistical analysis was calculated using Prism version 5.0 (GraphPad Software, USA). Data were analyzed using one- or two-way analysis of variance (ANOVA), presented as mean ± standard deviation (SD). All experiments were repeated three times, and the significant differences between groups were determined at *p* <0.05.

## Results

### Identification of the NCW Peptide from Clam Worm (*M. sanguinea*) Extract Using HPLC

**Fig. 1:**
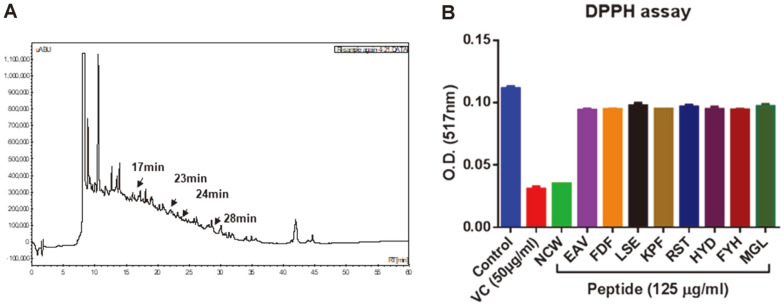
Identification of NCW peptide from clam worm extracts by high-pressure liquid chromatography (HPLC) and DPPH assay. (**A**) A typical HPLC of fraction of clam worm. (**B**) After incubation at 16°C for 15 min, the absorbance of final solution was measured at 517 mm using an ELISA microplate reader.

To identify the role of clam worm extract in antioxidant and anti-inflammatory activity, soluble extracts from clam worm were fractioned using HPLC and MS/MS analysis was used to analyze the peptide sequence. As shown in Fig. 1A, the retention time of clam worm extract was about 23 min and we identified nine peptides from substrate including NCW, EAV, FDF, LSE, KPF, RST, HYD, FYH, and MGL (Table 1). To determine the antioxidant effects of nine peptides, we performed 2,2-diphenyl-1-picrylhydrazyl (DPPH) assays. The DPPH radical scavenging activity of peptides was compared with that of vitamin C, another antioxidant. The result showed that the radical scavenging activity of NCW peptide was found to be highest in 9 peptides, similar with vitamin C (Fig. 1B). Therefore, we selected NCW peptide for the following study.

### Effects of NCW on the Activities of Antioxidant Enzymes

**Fig. 2:**
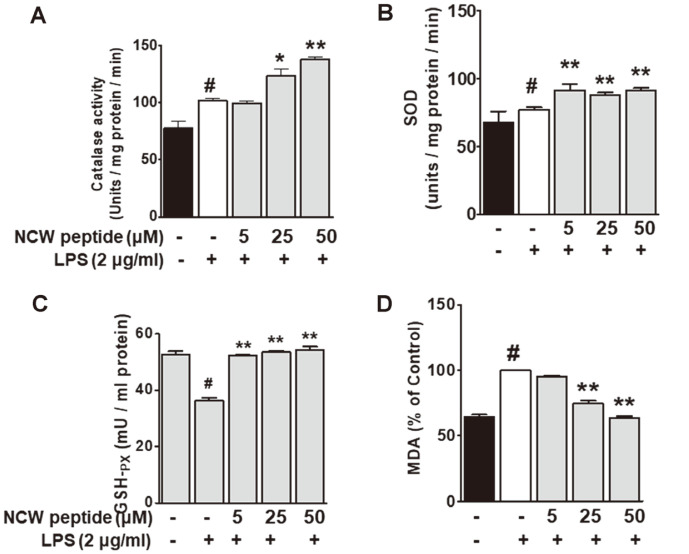
Effect of NCW peptide on antioxidant enzyme activities in LPS-stimulated RAW264.7 cells. Cells were pre-incubated with various NCW peptide for 2 h and then incubated with 2 μg/ml of NCW peptide for 20 h. The level of pro- inflammatory cytokines, (**A**) catalase (CAT), (**B**) superoxide dismutase (SOD), (**C**) glutathione peroxidase (GSH-Px), and (**D**) MDA were analyzed using cell supernatant. Each value represents mean ± SD. *p < 0.05 as compared with the LPS treatment group (#).

The antioxidant activity of NCW peptide can be considered by its capacity to scavenge stable DPPH free radical. In our preliminary data, it was found that NCW peptide showed a high DPPH radical scavenging activity (IC_50_ of 17.48 μg/ml) compared to vitamin C (2.752 μg/ml). Biochemical parameters including CAT, SOD, GSH-Px, and MDA levels were examined to further elucidate the antioxidant mechanism of NCW peptide. CAT activity was markedly higher in the 25-50 μM concentrations of NCW peptide than LPS-treated group (Fig. 2A). Next, SOD activity was significantly enhanced in the 5-50 μM concentrations of NCW peptide when compared with the LPS- treated group (Fig. 2B). Moreover, GSH-Px activity was markedly increased in the 5-50 μM concentrations of NCW relative to activity in the LPS-treated group (Fig. 2C). MDA levels in LPS-stimulated RAW264.7 were also significantly increased in the control (*p* < 0.05) whereas they decreased significantly upon treatment with NCW at the 5-50 μM concentrations (*p* < 0.05) (Fig. 2D). These results indicate that the response to treatment with high doses (25 and 50 μM) of NCW peptide is more potent than the response to low concentrations (5 μM), corresponding with higher catalase activity.

### Effect on Cell Cytotoxicity of NCW Peptide on RAW264.7 Cells

**Fig. 3:**
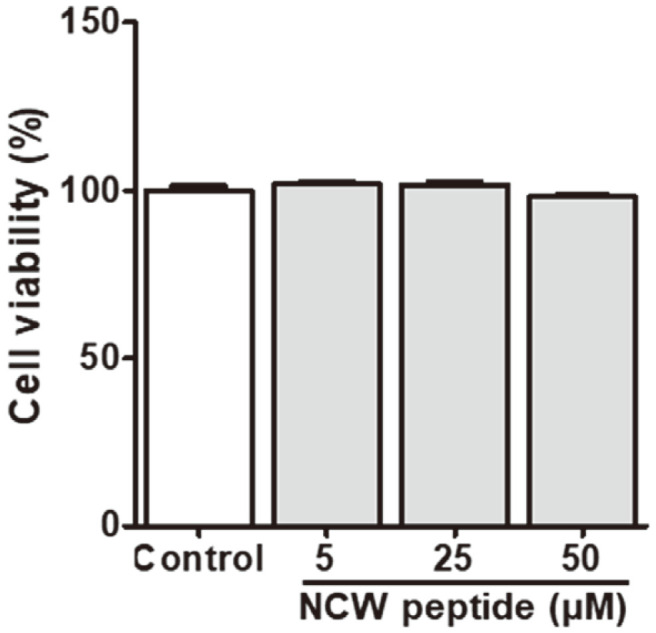
Cytotoxic effect of NCW peptide on RAW264.7 cells. Cells were treated with or without the NCW peptide at 5, 25, and 50 μM for 24 h, and the cell viability was determined by optimal density at 450 nm using the MTT assay. Values represent the mean and standard deviation (SD) of triplicate experiments (*n* = 3).

The cytotoxic activity of NCW peptide on RAW264.7 cells was analyzed by exposing the cells to various concentrations (0, 5, 25, and 50 μM) of NCW peptide for 24 h. According to our results, NCW peptide had no significant cytotoxic effects on RAW264.7 cells at the indicated concentrations compared with control (Fig. 3).

### Effects of NCW Peptide on Nitrite Production

**Fig. 4:**
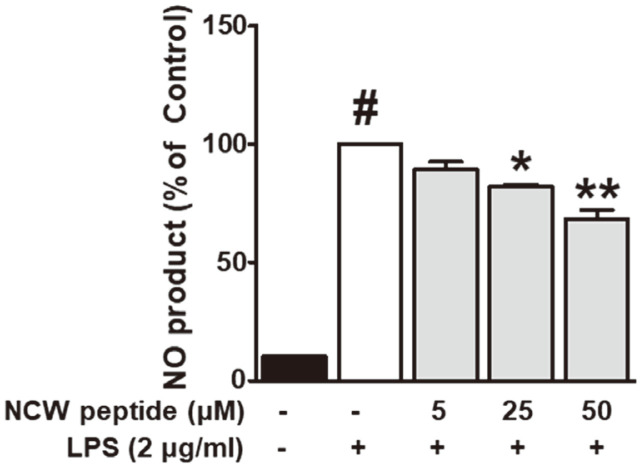
Effects of NCW peptide on nitrite production in LPS-induced RAW264.7 macrophages. The level of nitrite (NO) was detected using a Griess reaction assay. RAW264.7 cells were pre-treated with various concentrations of NCW peptide for 30 min and then incubated with 2 μg/ml of LPS for 20 h. *p < 0.05 as compared with the LPS alone group (#).

To investigate the anti-inflammatory effect of NCW peptide, we performed the NO assay in the supernatant of RAW264.7 cells. We pretreated cells with or without the NCW peptide (5-50 μM) for 30 min before stimulation with 2 μg/ml LPS for 24 h. As shown in Fig. 4, LPS treatment increased NO production approximately eight-fold, whereas treatment with NCW markedly reduced LPS-stimulated NO production in a concentration-related manner. These results suggest that NCW peptide might have anti-inflammatory activity in LPS-stimulated RAW264.7 cells.

### Effect of NCW Peptide on Activity Pro-Inflammatory Cytokines and Mediators in LPS-Induced RAW264.7 Cells

**Fig. 5:**
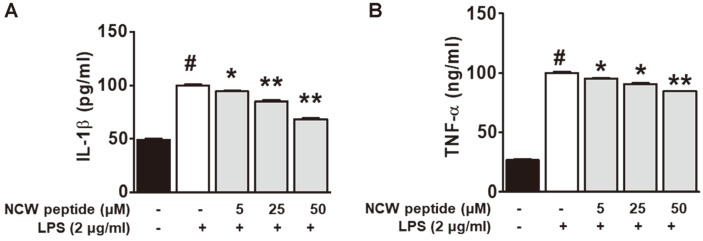
Effect of NCW peptide on IL-1β and TNF-α level in LPS-stimulated macrophage RAW264.7 cells. RAW264.7 cells were pretreated with or without of NCW peptide at 5, 25, and 50 μM for 30 min and then incubated with 2 μg/ml of LPS for 24 h (**A**) and 8 h (**B**). The levels of IL-1β and TNF-α were determined by ELISA microplate reader according to the manufacturer’s instructions. *p < 0.05 as compared with the LPS alone group (#).

To investigate the anti-inflammatory effect of NCW peptide in vitro, RAW264.7 cells were exposed to NCW peptide or LPS and then the quantities of IL-1β and TNF-α were measured in the cultured medium. NCW peptide markedly inhibited the LPS-stimulated IL-1β production in a concentration-dependent manner compared with the LPS-alone group (Fig. 5A). Moreover, NCW peptide significantly blocked the generation of TNF-α in indicated concentrations of NCW peptide compared with LPS-induced RAW264.7 cells (Fig. 5B). These results strongly suggest that NCW peptide can inhibit the secretion of pro-inflammatory cytokines IL-1β and TNF-α in LPS-stimulated RAW264.7 cells.

### Effect of NCW Peptide on iNOS and COX-2 Expression in LPS-Stimulated Macrophage RAW264.7 Cells

**Fig. 6:**
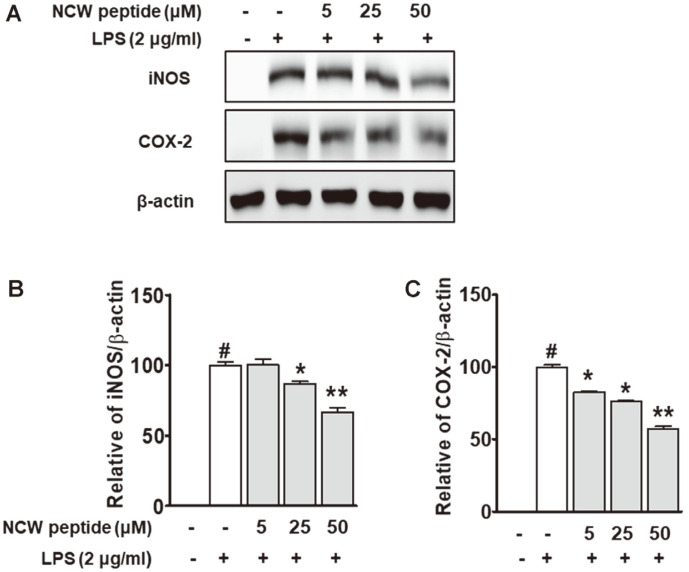
Effects of NCW peptide on iNOS and COX-2 protein expression in LPS-stimulated RAW264.7 cells. (**A**) The protein expression levels of iNOS, COX-2, and β-actin were detected using immunoblot analysis against specific antibodies. Quantification of protein levels was normalized to β-actin as loading control using a densitometer (Bio-Rad) (**B** and **C**). *p < 0.05 as compared with the LPS-alone group (#).

To find out whether the inhibition of NO generation was caused by the reduced iNOS activity, we examined the expression of iNOS and COX-2 protein by immunoblot analysis. As shown in Fig. 6A, treatment with LPS increased protein expression of iNOS and COX-2 in RAW264.7 cells. However, the increased levels of iNOS and COX-2 were reversed by treatment with NCW peptide at 5-50 μM compared with the LPS-alone group. We also quantitated the intensity of the protein levels using a densitometer in three independent experiments. The NCW peptide significantly decreased both iNOS and COX-2 proteins in LPS-induced RAW264.7 cells (Figs. 6B and 6C). These results implied that NCW peptide can reduce expressions of iNOS and COX-2 level in LPS-induced RAW264.7 cells.

## Discussion

Extractions and uses of biological compounds from earthworm have been traditionally utilized in various countries such as China, India, Myanmar, Korea, and Vietnam [16]. There are numerous studies on the anti- oxidant and anti-inflammation activities of extracts from earthworm [7, 8], although very few studies have been done on the sources from clam worm (*M. sanguinea*). In our previous studies, the extract of clam worm was reported to decrease activities of inflammatory cytokines (TNF-α, and IL-1β), to induce activities of antioxidant enzymes (CAT, SOD and GSH-Px) and to decrease the level of MDA in treatment with peptidoglycan [10]. The extract also exhibited better antioxidant and anti-inflammatory effects than those from earthworm in LPS- stimulated RAW264.7 cells [11]. Subsequently, we analyzed the extract of clam worm by using HPLC and the full amino acid sequence was obtained from MS/MS (Table 1) NCW peptide consists of 16 amino acids, N′- NCWPFQGVPLGFQAPP-C′, a novel peptide that had not been reported yet.

Reactive oxygen species (ROS) can produce activated oxygen such as hydrogen peroxide (H_2_O_2_), singlet oxygen, hydroxyl radical, and superoxide anion [17]. Over-production of ROS can generate DNA damage, enzyme inactivation, cellular necrosis and apoptosis, and lipid peroxidation, which are related with other pathological diseases including inflammation [18]. Therefore, antioxidants are important molecules that offer protection against the harmful effects of ROS [19].

Pigeolet and colleagues reported that the activities of antioxidant enzymes including CAT, SOD, and GSH-Px, are the most important enzymes for cells in defending against oxidative stress and are essential regulators on detoxification of O^2-^ [20, 21]. Endogenous GSH is a known oxy-radical scavenger and is suggested to play a central function against LPS-stimulated inflammatory response. In addition, MDA production takes place by free radical attacks on the plasma membrane and LPS-stimulated inflammation may lead to the accumulation of MDA. Thus, increased GSH levels lead to reduced MDA generation [22]. In the present study, NCW peptide significantly increased the activity of cellular antioxidant enzymes such as CAT, SOD, and GSH-Px and inhibited the level of MDA in LPS-induced RAW264.7 cells. These results suggest that NCW peptide possesses considerable antioxidant activity and the reduction of MDA may result from the activation of CAT, SOD, and GSH-Px level.

LPS can lead to the generation of pro-inflammatory cytokines, including TNF-α, IL-1β, and NO, as well as activate iNOS and oxidants [23]. Over-production of TNF-α, IL-1β, and NO plays a central role in chronic inflammatory diseases [24]. Inhibition of the production of inflammatory cytokines and mediators plays as a key role in controlling inflammation, and the suppression of their expression has the therapeutic potential to treat inflammatory diseases [25].

Our findings suggest that the novel peptide, NCW peptide, has antioxidant and anti-inflammation effects by reducing the pre-inflammation cytokines (iNOS, COX-2, TNF-α, and IL-1β) and by increasing the activities of antioxidant enzymes such as CAT, SOD and GSH-Px, and by decreasing the level of MDA. Thus, NCW peptide may be beneficial to human health due to its potential therapeutic application as a novel anti-inflammatory source.

## Figures and Tables

**Table 1:** Identification of peptide using MS/MS.

Fraction	Charge	m/z	Sequencing	Calc. mass
17 min	2	562.78	MGLVSDLNLY	1124.56
	2	632.32	RSTSSLDTRKL	1263.64
	3	352.17	FYHGDLRF	1054.51
	2	505.31	EAVLHKSLL	1009.62
	2	604.79	HYDLFNRFP	1208.58
23 min	2	879.43	NCWPFQGVPLGFQAPP	1757.86
24 min	3	703.85	FDFGQPTALFYT	1406.7
	2	933.47	KPFSKPALYESEEFPP	1865.95
28 min	2	932.98	LSENLLLAALLTPPPAMT	1864.96

Values are presented as mean±standard deviation.
